# Induction of targeted necrosis with HER2-targeted platinum(iv) anticancer prodrugs†
†Electronic supplementary information (ESI) available: Preparation and characterization of Pt(iv)–peptide conjugates and *in vitro* experiments. See DOI: 10.1039/c5sc00015g



**DOI:** 10.1039/c5sc00015g

**Published:** 2015-03-16

**Authors:** Daniel Yuan Qiang Wong, Jun Han Lim, Wee Han Ang

**Affiliations:** a Department of Chemistry , National University of Singapore , Singapore 117543 , Singapore . Email: chmawh@nus.edu.sg ; Tel: +65 6516 5131; b NUS Graduate School for Integrative Sciences and Engineering , Centre for Life Sciences (CeLS) , Singapore 117456 , Singapore

## Abstract

It is well-recognized that the failure of many chemotherapeutics arises due to an inability to induce apoptosis. Most cancers acquire a myriad of pro-survival adaptations, and the vast heterogeneity and accumulation of multiple often unrelated anti-apoptotic signaling pathways have been a major stumbling block towards the development of conventional chemotherapeutics, which can overcome drug resistance. We have developed highly potent and selective HER2-targeted Pt(iv) prodrugs bearing anti-HER2/neu peptides that induce *targeted necrosis* as a novel strategy to circumvent apoptosis-resistance. These Pt(iv)–peptide conjugates exhibit a unique biphasic mode of cytotoxicity comprising rapid killing of cancer cells *via* necrosis in the first phase followed by an extended and gradual phase of delayed cell death. We demonstrate that these Pt(iv)–peptide prodrugs are more potent than their Pt(ii) congeners in direct cell-killing and exhibit comparable long-term inhibition of proliferative capacity and with greater selectivity against HER2-positive cancer cells.

## Abstract 2

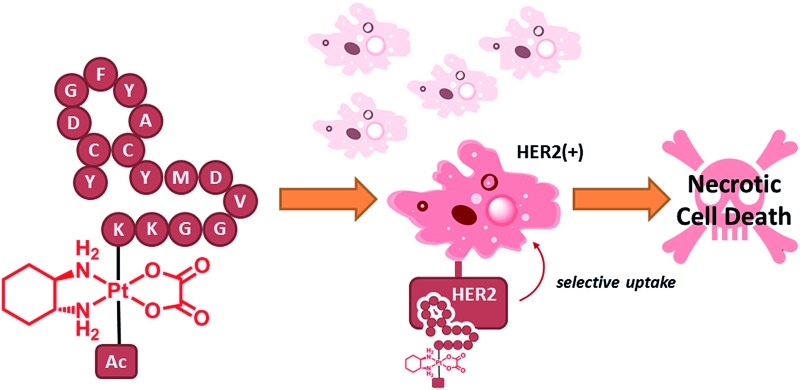
Platinum(iv) prodrug complexes based on the cisplatin/oxaliplatin pharmacophore, containing anti-HER2/neu targeting peptides, were designed to deliver their cytotoxic platinum(ii) payload selectively to highly HER2-expressing cells. Through induction of necrotic cell death, these platinum(iv)–peptide conjugates can circumvent apoptosis-resistance pathways in targeted HER2-positive cells.

## Introduction

Molecularly-targeted chemotherapeutics have made considerable progress in the clinical outcomes for many malignancies.^1^ The recognition that certain molecular pathways are critical to carcinogenesis and continued tumour progression, and may therefore represent an Achilles' heel, has triggered a revolution in cancer drug development. However, drug resistance continues to be a significant challenge and it is well-recognized that the failure of many chemotherapeutics arises due to an inability to induce apoptosis at a cellular level.^2^ Most cancers progressively acquire a myriad of pro-survival adaptations such as a mutated p53 tumor suppressor gene and upregulation of the anti-apoptotic Bcl-2 signal, which render them refractory to treatment. Although a number of experimental strategies to restore apoptosis sensitivity have been explored, these approaches have been severely challenged by the vast heterogeneity within the same tumor mass and the accumulation of multiple unrelated pro-survival mutations simultaneously, which frustrate molecularly-targeted approaches.^3^ Conversely, necrotic cell death has traditionally been shunned in favor of apoptosis because the abrupt release of intracellular contents in the extracellular space triggers an unintended inflammatory response. Yet it has been recognized in recent years as a regulated mode of cell death, distinct from apoptosis, which may be harnessed to circumvent defects in apoptotic signaling pathways.^4^

The HER2 receptor tyrosine kinase is a clinically-validated molecular target responsible for the initiation, progression and metastasis of a variety of cancers.^5^ It is an epidermal growth factor receptor, which is highly over-expressed on the cell surface of around 20–30% of breast^5^ and 20% of gastric cancers^6^ and has been associated with poorer prognosis. The first FDA-approved clinical agent to exploit HER2 over-expression is the monoclonal anti-HER2/neu antibody trastuzumab (Herceptin®). It has been proposed that trastuzumab antagonizes the downstream growth signaling pathway of HER2/neu by inhibiting HER2 dimerization as well as promoting immune-mediated killing through antibody-dependent cellular cytotoxicity.^7^ In contrast to the full-length antibody (*ca.* 150 kDa), a shorter peptidomimetic could retain comparable targeting capability while displaying superior tissue penetration. A family of short exocyclic peptides designed to mimic the CDR-H3 recognition loop of trastuzumab, named AHNP (anti-HER2/neu peptide), have been shown to bind HER2 with high affinity.^8^

**Fig. 1:**
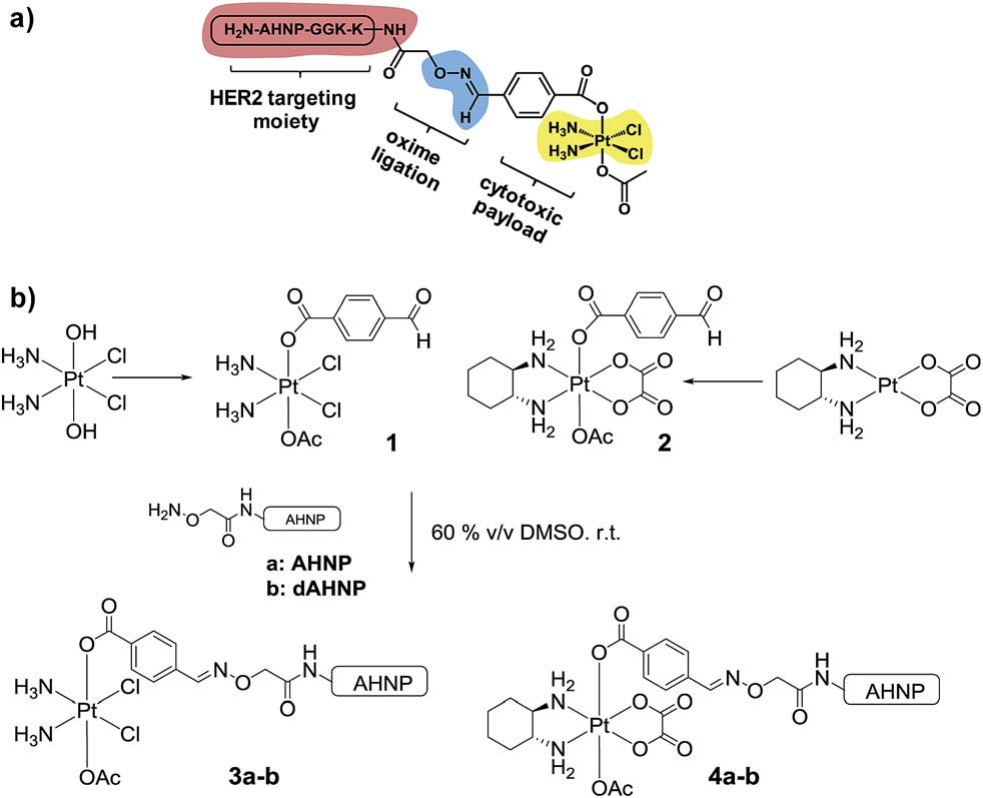
Synthesis of HER2-targeted Pt(iv)–AHNP conjugate consisting of an AHNP motif tethered to a cytotoxic Pt pharmacophore. The AHNP motif was separated with a small tripeptide spacer (GGK) and functionalized with an aminooxy(acetate) linker at the terminal lysine residue. AHNP sequence: H_2_N-YC*DGFYAC*YMDVGGKK (aminooxy)-CONH_2_ (* – linked disulfide bridge).

In order to achieve highly selective Pt drugs, we developed a HER2-targeted Pt agent by tethering an anti-HER2/neu peptide (AHNP) targeting sequence to Pt(iv) prodrug scaffolds of cisplatin (CDDP) or oxaliplatin (OXP) *via* chemoselective oxime ligation (Fig. 1a).^9^ The Pt(iv) scaffolds are native prodrugs, which can be activated by intracellular reduction to release their cytotoxic Pt(ii) payload with concomitant dissociation of the axial ligands.^10^ General considerations for the design of Pt(iv) prodrugs have been recently reviewed.^11^

Here in this work, we report that Pt(iv)–AHNP conjugates exhibit a unique biphasic mode of action, which is profoundly different from their clinically used Pt(ii) counterparts. The first phase involves rapid killing *via* targeted necrosis instead of apoptosis, due to massive Pt influx. The surviving cell population, although still viable, exhibited a phenomenon of delayed cell death, possibly due to mitotic catastrophe. The Pt(iv)–AHNP conjugates were also more selective against HER2-amplified cells while sparing normal HER2 expressing cells. Although the concept of targeted necrosis has often been advocated, there have been very few actual proof of concepts reported in the literature.^4*b*^ We offer evidence supporting targeted necrosis as a viable alternative to conventional apoptosis-inducing agents, while precluding potential non-specificity associated with the necrotic death pathway.

## Results and discussion

### Design and synthesis of HER2-targeted Pt(iv)–AHNP conjugates

We postulated that a Pt(iv) prodrug scaffold conjugated to the HER2-targeting AHNP peptide could selectively deliver CDDP or OXP into HER2 over-expressing cells while reducing collateral toxicity. We previously showed that the asymmetrical Pt(iv)–benzaldehyde-bearing scaffold, based on CDDP, was reduced with dissociation of the axial ligands to yield CDDP predominately.^12^ The CDDP-based Pt(iv) scaffold **1** was synthesized as previously described starting from oxoplatin, by reacting with a *N*-hydroxysuccinimide-ester of 4-formylbenzoic acid followed by acetylation with acetic anhydride (Fig. 1b).^12^ The OXP-based Pt(iv) scaffold **2** was synthesized *via* a different route in order to take advantage of the synthetically accessible *trans*-OXP–(OH)(OAc) precursor^13^ by acylation with 4-formylbenzoyl chloride in the presence of pyridine as an organic base. The crude product was subsequently purified by flash column chromatography. Acetal formation due to the reactive aldehyde moiety was readily observed in the presence of MeOH and can be resolved by excluding it entirely. Pt(iv)–AHNP conjugates, based on CDDP and OXP, **3a** and **4a** were synthesized *via* a chemoselective oxime ligation strategy.^9,12^ Pt(iv)–dAHNP conjugates **3b** and **4b**, bearing a non-binding peptide altered at 3 key residues on ANHP, were prepared as controls. In general, these Pt(iv)–peptide conjugates were prepared by treating the desired (aminooxy)acetylated peptide with a slight stoichiometric deficit of **1** or **2** in 60% v/v DMSO. The oxime ligation proceeded quickly and was usually completed within 2 h, with minimal side-products observed. The crude products were purified by semi-preparative HPLC with moderate yields between 19–51% after isolation. Characterization of the conjugates was carried out using ESI-MS and their purities were ascertained to be between 94–98% purity by analytical HPLC (see ESI†).

### Pt(iv)–AHNP conjugates exhibit rapid and massive drug uptake and selectively kill HER2(+) cells in co-culture

**Fig. 2:**
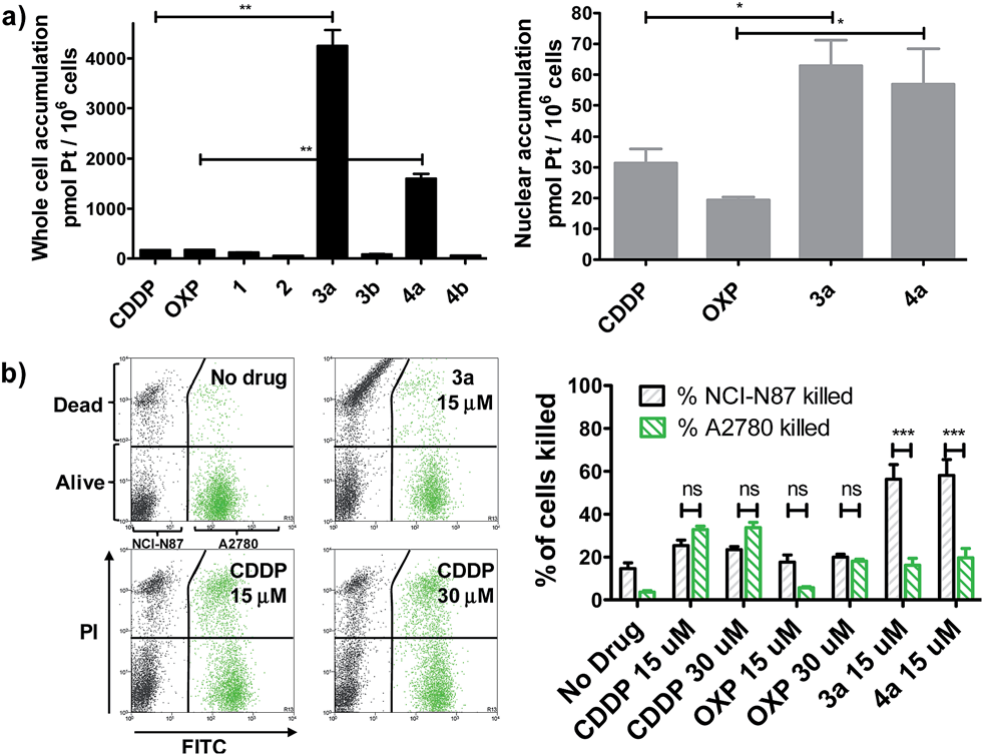
(a) Pt(iv)–AHNP conjugates are much more efficiently taken up into cells. Whole cell (left) and nuclear (right) uptake after drug treatment (20 μM) for 4 h in NCI-N87 as measured by ICP-MS. Statistical analysis by unpaired Student's *t* test. (b) Pt(iv)–AHNP conjugates selectively kill HER2 over-expressing NCI-N87 over the normal HER2 expressing A2780. A co-culture of NCI-N87 and A2780 (pre-stained with CellTracker™ Green) was drug-treated for 24 h before viability staining with PI. Statistical analysis by two-way ANOVA and Bonferroni post-tests. Means ± s.e.m. (*p < 0.05, **p < 0.01, ***p < 0.001).

We evaluated whether Pt(iv)–AHNP could enhance cellular uptake and deliver their cytotoxic payload through the HER2-targeting motif in line with our design strategy. Enhanced uptake of **3a** and **4a** was observed, following short-term drug exposure to the highly HER2-expressing NCI-N87 gastric cancer cell-line for 4 h and the subsequent analysis of whole cell and nuclear uptake by ICP-MS (Fig. 2a).^14^ Although extracellular reduction of Pt(iv) in cell-culture media may occur, this was not expected to be a significant factor within the short 4 h duration.^15^ CDDP and OXP exhibited similar whole cell uptakes of 170.2 ± 3.5 and 170.1 ± 6.4 pmol per 10^6^ cells, respectively. The targeted CDDP–AHNP (**3a**) and OXP–AHNP (**4a**) conjugates presented dramatically higher whole-cell uptakes of 4246 ± 450 and 1598 ± 138 pmol per 10^6^ cells, respectively, which was *ca.* 25-fold and 9-fold greater than their parental drug (*p* < 0.01). Accordingly, the nuclear uptake of **3a** and **4a** was 3-fold and 12-fold higher compared to CDDP and OXP, respectively (*p* < 0.05). In contrast, the untargeted Pt(iv) precursors, **1** and **2**, as well as the control Pt(iv)–dAHNP conjugates, **3b** and **4b**, were not as readily taken into NCI-N87. Since the control peptide dAHNP differed minimally from AHNP, mainly by substitution of l to d-isomer amino acids, and it is expected to have similar polarity, the superior uptake of **3a** and **4a** suggests selective uptake mediated by HER2 receptors, rather than by passive diffusion. In agreement, both **3a** and **4a** were cytotoxic while the control conjugates **3b** and **4b** were effectively non-cytotoxic (IC_50_ > 100 μM) based on MTT metabolic activity assays after 72 h exposure (Fig. S2†). We reasoned that the massive and rapid uptake of the targeted Pt agents relative to their parental drugs within a short time frame could explain the conjugates' unique killing profile.

In line with our hypothesis of selective targeting, we further evaluated whether **3a** and **4a** could selectively kill HER2 positive cells while sparing cells with basal HER2 levels. We utilized a co-culture model comprising NCI-N87, with high HER2 expression, and A2780 ovarian carcinoma, with significantly lower but still detectable levels of HER2.^16^ A co-culture of NCI-N87 (unstained) and A2780 (pre-stained with CellTracker™ Green as a tracer) was drug-treated for 24 h before staining with propidium iodide (PI) to determine cell viability by flow cytometry analysis. A ratio of 4 : 1 NCI-N87 (48 h doubling time) to A2780 (13 h doubling time) was seeded 24 h prior to drug treatment so that both cell lines were at approximately equal proportions at the point of treatment, as estimated by flow cytometry analysis. The Pt-sensitive A2780 ovarian cancer cell-line was chosen rather than healthy primary cultures as a more challenging co-culture model, because both CDDP and OXP already feature a modestly favourable therapeutic index, killing cancer cells over normal healthy cells in most cases. The results indicate that **3a** and **4a** not only demonstrated superior killing of NCI-N87 but were more selective compared to the untargeted CDDP and OXP (*p* < 0.001) (Fig. 2b). CDDP and OXP treatment of the co-culture did not discriminate between NCI-N87 and A2780. In contrast, the trend was sharply reversed with **3a** and **4a** which killed a significantly higher percentage of NCI-N87 relative to A2780 (*p* < 0.001).

### Distinctly different cell-killing profile of Pt(iv)–ANHP conjugates *via* targeted necrosis

**Fig. 3:**
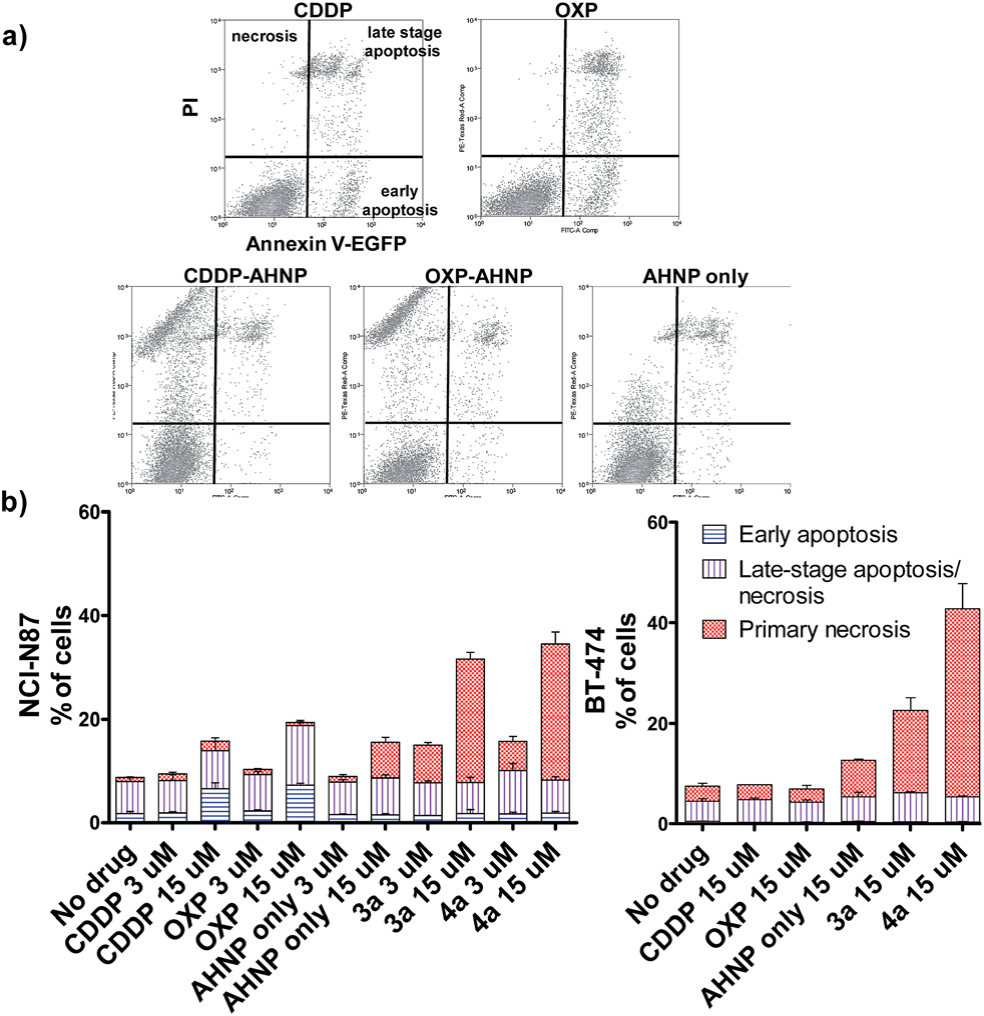
Apoptosis was evaluated by Annexin V/PI staining of drug-treated cells after 24 h. (a) Scatter plot of treated NCI-N87 cells (15 μM drug) (b) histograms of apoptosis-sensitive NCI-N87 (left) and apoptosis-resistant BT-474 (right) after 24 h exposure.

Clinical Pt(ii) agents, namely CDDP, carboplatin and OXP, induce tumor cell death through DNA-damage mediated apoptosis.^2b,17^ CDDP was also reported to induce necrosis *in vitro* at very high concentrations (*ca.* 100 × IC_50_).^18^ We therefore employed the Annexin V apoptosis assay in order to evaluate the mode of cell death induced by Pt(iv)–AHNP conjugates **3a** and **4a**. Cells were exposed to the Pt agents for 24 h before staining with Annexin V-EGFP and PI. Annexin V binds to surface-exposed phosphatidylserine residues, which migrate from the inner plasma membrane leaflet towards the outer membrane during apoptosis.^19^ To facilitate comparison, the complexes were tested at equi-concentrations (15 μM), close to the clinically relevant peak plasma CDDP concentration of 13.03 ± 4.70 μM after a standard intravenous dosage (100 mg m^–2^).^20^ As expected, both CDDP and OXP-treated NCI-N87 cells displayed appreciable apoptotic cell populations in early (Annexin V+/PI–) and late-stage apoptosis (Annexin V+/PI+), which increased in a dose-dependent manner (Fig. 3a and b(left)). In stark contrast, both **3a** and **4a**-treated NCI-N87 show predominately cell death *via* primary necrosis (Annexin V–/PI+), indicating that the plasma membrane has been compromised without phosphatidylserine exposure (Fig. 3a and b(left)).

**Fig. 4:**
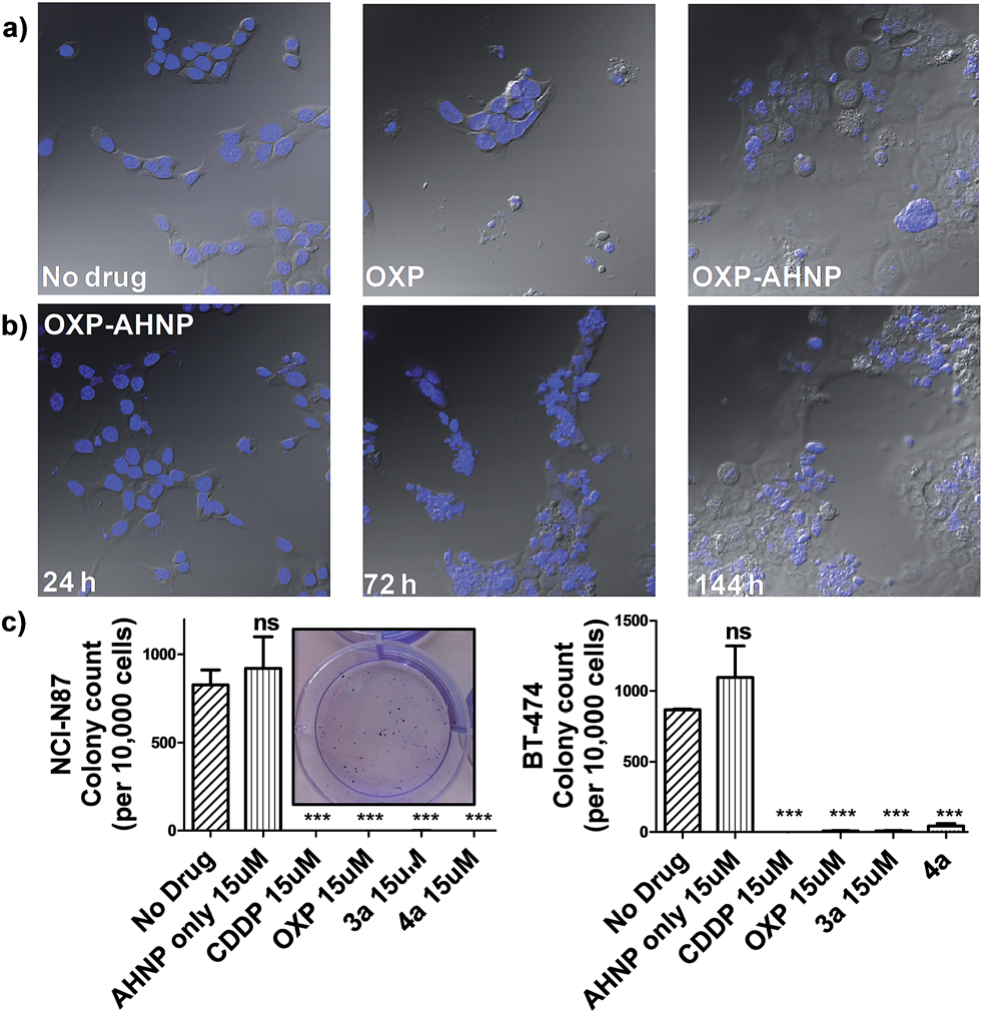
(a) Representative overlaid microscopy images of the control and the treated NCI-N87 (15 μM drug for 24 h and allowed to recover in fresh media for a week). Differences in nuclear morphology were visualized by Hoechst 3342 staining. Untreated cells were shown at day 1 while drug-treated cells were shown at day 6. (b) **4a**-treated NCI-N87 undergoes short-term proliferation 24 h after drug treatment but displays increasingly severe nuclear fragmentation over 7 d (left to right). (c) Clonogenic assay of NCI-N87 (left) and BT-474 (right) to assess the long-term proliferation ability of single cells drug-treated for 24 h and allowed to recover in fresh complete media. Representative image of AHNP-treated NCI-N87 colonies shown in the inset. Statistical analysis by unpaired Student's *t* test against non-treated control. Means ± s.e.m. (***p < 0.001).

In agreement, the morphology of the OXP-treated NCI-N87 cells displayed characteristic features of apoptotic cell death (cell rounding and shrinkage, blebbing of plasma membrane, condensation and fragmentation of DNA) (Fig. 4a).^21^ On the other hand, **4a**-treated NCI-N87 did not show the same defining morphology and instead presented irregular nuclear fragmentation (both karyolysis and karyorrhexis) as well as the appearance of dead de-nucleated cell-debris, still attached to the substrate, bearing the same polygonal morphology as healthy cells. The exposure of phosphatidylserine is an early event in the apoptotic process, which precedes many other characteristic features such as cell-shrinkage and nuclear condensation.^19^ Although Pt(iv)–AHNP conjugates displayed higher nuclear Pt levels than their Pt(ii) congeners (Fig. 2a), therefore more DNA platination, only minimal apoptosis was observed in the former. This suggests that the Pt(iv)–AHNP conjugates induced very rapid membrane permeabilization before the apoptotic cascade of events could be initiated by DNA-damage recognition proteins.

As reactive oxygen species (ROS) is a well-known stimulus of necrosis, we further investigated if the observed necrosis was ROS-mediated. Cellular ROS on NCI-N87 was measured using 2′,7′-dichlorofluorescein diacetate (DCFDA), a redox-sensitive probe, 1 and 4 h after drug-treatment (Fig. S1†). After 4 h, **3a** and **4a** gave a 1.7-fold and 1.3-fold signal increase, respectively, over non-treated control cells. In comparison, the AHNP peptide alone led to a signal increase of 1.6-fold. Since the AHNP peptide was by itself a modest effector of ROS with associated necrotic cell death (Fig. 3b), a non-passive role of the targeting peptide in cell-killing cannot be dismissed. Nonetheless, ROS levels of **3a** and **4a**-treated cells were not correlated with their cell-killing ability and it is thus probable that ROS was not the sole factor in inducing necrotic cell death here.

The loss of a functional p53 is a well-established biomarker for Pt-associated apoptotic resistance both *in vitro* and *in vivo*, which arises due to consequent failure in enacting the apoptotic cell death machinery in spite of DNA damage.^2b,17^ In particular, BT-474 breast ductal carcinoma is resistant to apoptosis on the basis of its temperature-sensitive p53 status that is defective at 37 °C.^22^ In keeping with NCI-N87, BT-474 cells exhibit high HER2 expression levels.^23^ The Pt(iv)–AHNP conjugates were more potent in both apoptosis-sensitive NCI-N87 and apoptosis-resistant BT-474 cells compared to their parental drug following 24 h exposure (Fig. 3b). The AHNP peptide alone displayed only modest cell-killing. In NCI-N87, about 31.6% and 34.5% of cells were non-viable (Annexin V+ or PI+) following 15 μM drug exposure of **3a** and **4a** compared to 15.8% and 18.8% with CDDP and OXP treatment, respectively. Against p53-dysfunctional BT-474 cells, no signs of early apoptosis (Annexin V+/PI–) were observed regardless of drug-treatment. Both CDDP and OXP-treated cells fared no better than untreated control cells (Fig. 3b). In contrast, the Pt(iv) conjugates **3a** and **4a** surpassed their parental drugs at 22.5% and 47.2%, respectively, through induction of necrosis. Taken together, these results suggest that **3a** and **4a** can overcome apoptotic resistance through HER-2 targeted necrosis.

### Surviving tumour cell population exhibits delayed cell death and suppression of long-term proliferation ability

We observed a curious biphasic killing mechanism on NCI-N87 cells by the Pt(iv)–AHNP conjugates, which was hinted at by visual monitoring of the drug-treated cells under conventional microscopy (Fig. 4b and S3†). The first phase comprised a rapid killing of between 30–35% of cells *via* necrosis (as ascertained by Annexin V), with continued proliferation of the surviving population followed by an extended and gradual phase of delayed cell death (Fig. S3†). This sharp discernible two-phase rate of killing was not apparent with the free parental drugs. The surviving population of Pt(iv)–AHNP treated cells were still viable and retained limited proliferation capacity in the short term (but not long term). The surviving population of the **3a** and **4a**-treated NCI-N87 cells were significantly more metabolically active compared to CDDP and OXP-treated cells after an intermediate 3 day period (Fig. S2†). The MTT assay, which measures oxidoreductase activity, was used to assess metabolic activity of treated cells relative to non-treated cells after 72 h. **3a** and **4a**-treated cells (15 μM) displayed higher metabolic activities of 48.2 ± 2.3% and 49.0 ± 2.1% compared to 25.0 ± 4.3% and 21.0 ± 4.3% with CDDP and OXP, respectively. This result suggested that there were more viable cells following treatment with **3a** and **4a** as compared to parental CDDP and OXP, despite the more rapid induction of necrotic cell death by the conjugates. Yet the MTT assay cannot distinguish between cell-killing and the inhibition of cell proliferation (cytostasis) since both these factors could contribute to the measured metabolic activities.

One of the hallmarks of cancer is the capacity for indefinite growth and replication.^24^ In order to track the long-term proliferation ability of the surviving cell population, we employed a combination of confocal microscopy and clonogenic assay to monitor the fate of treated cells (15 μM for 24 h), which were subsequently allowed to recover in fresh media for a span of time. Under microscopy monitoring, **4a**-treated NCI-N87 cells undergo modest cellular expansion but displayed increasingly severe nuclear fragmentation 1–6 d after treatment (Fig. 4b). The nuclear morphology at 24 h (when **4a** was aspirated) was still relatively intact, indicating that subsequent nuclear deformation was independent of the acute necrotic phase. We hypothesized that the abnormal nuclear morphology observed with concomitant delayed cell death was due to mitotic catastrophe, a consequence of aberrant mitosis.^4a,25^ **4a**-treated cells displayed micro-nucleation, multiple nuclei as well as enlarged irregular nuclei, which are morphological features suggestive of mitotic catastrophe (Fig. 4a and b).^4a,25^ **4a**-treated cells gave rise to abnormal progeny suggesting that the mitotic defect, presumably due to a combination of DNA damage and impairment to the mitotic machinery, was durable even after the compound was removed. By day 6, all **4a**-treated cells were either dead (de-nucleated) or exhibited severely abnormal nuclear morphology implying that continued long-term proliferation was unviable. On the contrary, the few surviving cell population in OXP-treated NCI-N87 displayed a healthy-looking nuclear morphology, which may suggest a capacity for re-growth if allowed to recover further (Fig. 4a). The cell cycle analysis of **4a**-treated NCI-N87 over 72 h indicated a transient G_0_/G_1_ arrest, which occurred between the 27th to 49th hour, followed by progression into the S phase and abrogation of the G_2_/M mitotic checkpoint between the 49th to 72nd hour (Fig. S4†). This mitotic entry of the cell despite cellular damage, and without an apoptotic response, is consistent with a hypothesis of genomic instability and mitotic catastrophe. On the other hand, OXP-treated cells indicated a markedly different DNA content profile, implying a different mechanistic action (Fig. S4†). There was a slight accumulation into the early S phase (but not late S phase), paralleled by a sharp increase in the sub-G_0_ phase (DNA laddering), which implied that cellular apoptosis was induced shortly after initiation of DNA synthesis.

Although the surviving population of **4a**-treated cells were more metabolically-active compared to OXP-treated cells and in fact exhibited transient cellular expansion, the progressively distorted nuclear morphology of the **4a**-treated cells and their progeny suggested that long-term cellular division was unlikely. Thus, a clonogenic assay was performed in order to substantiate this hypothesis. The clonogenic assay measures the ability of single cells to divide through at least six generations (forming colonies ≥50 cells). In both NCI-N87 and BT-474, pre-treating the cells for 24 h was sufficient to induce a near complete abolishment of colony forming units with CDDP, OXP and the Pt(iv)–AHNP conjugates (*p* < 0.001) but not with free AHNP (Fig. 4c and S5†). In NCI-N87, any significant differences between the free parental drug and conjugates **3a** and **4a** were not observed because both cell cycle arrest and cell death precluded colony formation. Furthermore, although the concentration tested for both CDDP and OXP was ineffective in triggering apoptosis in the resistant BT-474 cell-line, it was still sufficient to inhibit long-term proliferation. In contrast, free AHNP peptide was effective in directly killing both cell-lines (Fig. 3b) but was unable to halt long-term proliferation (Fig. 4c), indicating that these events are not necessarily correlated.

Taken together, our results indicate that the Pt(iv)–AHNP conjugates were mechanistically very different from their Pt(ii) counterparts, and of comparable potency but with greater selectivity.

## Conclusions

Unlike molecular targeted therapy, non-specific alkylating agents like CDDP and OXP may have multiple modes of action arising from different biological targets including DNA.^26^ It was reported that OXP-treatment could effect a tumor-specific immune response by triggering immunogenic cell death via endoplasmic reticulum stress.^27^ A broader spectrum of action could be therapeutically favorable because it potentially circumvents multi-factorial apoptosis-resistance signaling pathways. We investigated two potent HER2-targeted Pt(iv)–AHNP agents based on the clinically-used Pt(ii) drugs CDDP and OXP. Despite being prodrugs, the conjugation of a HER2-targeting peptide drastically altered the mode of cell death, presumably *via* massive and rapid Pt influx. These conjugates exhibited a unique biphasic mode of action and selectively killed highly HER2-expressing cells under co-culture conditions, even against phenotypes that are resistant to apoptosis. Our work highlights targeted necrosis as a viable alternative cell death modality that can be harnessed to overcome the defective apoptosis hampering many conventional chemo- and targeted anticancer agents.
