# Supplementary material for: Induction of targeted necrosis with HER2-targeted platinum(iv) anticancer prodrugs
Source: Chem Sci. 2015 Mar 16;6(5):3051–6. doi: 10.1039/c5sc00015g (PMC5490001; doi:10.1039/c5sc00015g)
Supplement: Supplementary file 1 [file SC-006-C5SC00015G-s001.pdf]

Electronic Supplementary Material (ESI) for Chemical Science.
This journal is © The Royal Society of Chemistry 2015

Supplementary Information

Induction of Targeted Necrosis with HER2-targeted Platinum(IV) Anticancer Prodrugs

Daniel Yuan Qiang Wong[ψ], Jun Han Lim[ψ], and Wee Han Ang*

[ψ] These authors contributed equally.

Table of Contents

Materials and Methods 2

Detailed Synthetic Scheme 8

Supplementary Figures

S1 – Measurements of cellular ROS after 1 and 4 h 9

S2 – Metabolic activity of drug-treated NCI-N87 after 3 days 10

S3 – Visual monitoring of drug-treated NCI-N87 11

S4 – Cell cycle analysis of drug-treated NCI-N87 12

S5 – Clonogenic assay of drug-treated NCI-N87 13

Characterisation of compounds 14

References 20

Materials and Methods

General reagents. Unless otherwise stated, all starting reagents are commercially available. Cisplatin,[4] *cis,cis,trans*-diamminedichloro(hydroxido)(4-formylbenzoate)platinum(IV) **1**,[1] oxaliplatin,[2] and *trans*-acetato[(1*R*,2*R*)-cyclohexane-1,2-diamine-*N*,*N'*](ethanedioato-*O*,*O'*) hydroxido-platinum(IV)[3] were synthesized as reported previously.

Peptides. The HER2/neu binding peptide (AHNP) and control non-binding peptide (dAHNP) were custom-synthesized by ChinaPeptides (Shanghai) and modified with an (aminooxy)acetic acid linker at the ε-amino group of the lysine residue as specified. The peptides AHNP and dAHNP has the following sequence: NH_2-*Tyr-Cys-Asp-Gly-Phe-Tyr-Ala-Cys-Tyr-Met-Asp-Val-Gly-Gly-Lys-Lys*(aminooxy)-$CONH_2$ and *D-Phe-Cys-Asp-Gly-Phe-Tyr-Ala-Cys-D-Tyr-Met-Asp-Val-Gly-Gly-Lys-Lys*(aminooxy)-$CONH_2$ respectively.

Instrumentation. ^{1}H NMR was recorded on a Bruker Avance 300 MHz or 400 MHz. Chemical shifts are reported in parts per million relative to residual solvent peaks.[4] Electrospray ionization mass spectra (ESI-MS) were obtained on a Thermo Finnigan LCQ ESI-MS system. Elemental analysis was carried out on a Perkin-Elmer PE 2400 elemental analyzer by CMMAC (National University of Singapore). Platinum concentrations of stock solutions were measured externally by CMMAC (National University of Singapore) on an Optima ICP-OES (Perkin-Elmer). In some cases, platinum concentrations were also measured on an Agilent 7500 Series ICP-MS. Analytical UV-vis absorbance was measured on a Shimadzu UV-1800 UV-vis spectrophotometer. Analytical reversed phase HPLC (RPLC) was conducted on a Shimadzu Prominence or an Agilent 1200 series DAD using Shimpack VP-ODS column (5 μm, 120 Å, 150 × 4.60 mm, 1.0 mL min^{-1} flow). Semi-preparative HPLC was performed on a Shimadzu Prominence using YMC-Pack Pro C18 column (5 μm, 120 Å, 250 × 10 mm, 2.0 mL min^{-1} flow).

Synthesis of *trans*-acetato[(1*R*,2*R*)-cyclohexane-1,2-diamine](ethanedioato-*O*,*O'*)(4-formylbenzoate)platinum(IV) (2). 4-formyl-benzoyl chloride (111.8 mg, 0.663 mmol) dissolved in dry acetone (2 mL) was added dropwise to a suspension of *trans*-acetato[(1*R*,2*R*)-cyclohexane-1,2-diamine-*N*,*N'*](ethanedioato-*O*,*O'*)hydroxidoplatinum(IV) (105.1 mg, 0.222 mmol) and pyridine (680 μL, 8.44 mmol) in dry acetone (15 mL). The reaction mixture was

stirred vigorously and refluxed overnight to give a pale yellow suspension. The solvent was evaporated *in vacuo* and the precipitate was dissolved in minimal MeCN and precipitated with chilled 1:1 DCM/diethyl ether. The crude mixture was left at 4 ^{o}C for 1 h to promote further precipitation. The crude precipitate was then triturated with DCM, redissolved in acetonitrile and purified by flash silica gel chromatography (1:1 DCM/MeCN and 0.01% AcOH; R_f = 0.4) to yield a yellow powder. Yield: 14.2 mg (10.6 %) ^{1}H NMR (DMSO-d_6, 300.13 Hz): δ 10.08 (s, 1H), 8.4 (br m, 4H), 8.05 (d, 2H), 7.97 (d, 2H), 2.66 (br m, 2H), 2.16 (m, 2H), 1.99 (s, 3H), 1.52 (m, 4H), 1.11 (m, 2H); ESI-MS (-ve): m/z 604.0 $[M-H]^-$; $C_{18}H_{22}N_2O_9Pt.2H_2O$ (641.5 g/mol) calc: C 33.70, H 4.09, N 4.37; found C 33.36, H 3.91, N 4.38. Purity (HPLC): 1 peak at 254 and 280 nm.

General procedure for synthesis of platinum(IV)-peptide conjugates. In general, the platinum(IV)-peptide conjugates (**3a-b** and **4a-b**) were prepared by treating the desired (aminooxy)acetylated peptide with slight stoichiometric deficit of **1** or **2** in 60 % v/v DMSO. All reagents were pre-dissolved in DMSO to form stock solutions. Concentrations of the free peptide was determined by UV at 280 nm either in 50 mM pH 7 phosphate buffer or 1 % aq. ammonia.[5] Reaction was generally completed within 2 – 4 h as determined by analytical HPLC. The desired products were subsequently isolated by semi-preparative HPLC using a gradient elution of 10 – 30 % solvent B for the first 25 min followed by 30 % B for another 17 min. Purity of the conjugates was assessed on an analytical column assessed using a gradient elution of 8 – 30 % B for the first 10 min, 30 % B for another 8 min and finally 30 – 80 % B for 7 min. Solvent A is aq. NH_4OAc buffer (10 mM, pH 7) and solvent B is MeCN.

Cisplatin(IV)-AHNP conjugate (3a). Complex **1** (50.5 uL of a 60.19 mM stock solution, 3.04 μmol) was added to aminooxy-functionalized AHNP peptide (150 μL of a 23.76 mM stock solution, 3.56 μmol) in 1.4 mL 60 % v/v DMSO. The reaction mixture was agitated for 2 h before purification by semi-preparative HPLC. Yield: ca.1.5 mg (21 %); ESI-MS(-): m/z 1188.6 $[M - 2H]^{2-}$; Purity (HPLC): 98 % at 280 nm.

Cisplatin(IV)-dAHNP conjugate (3b). Synthesis of **3b** was similar to **3a**. Yield: ca. 43 %; ESI-MS(-): m/z 1180.9 $[M – 2H]^{2-}$; Purity (HPLC): 97 % at 280 nm.

Oxaliplatin(IV)-AHNP conjugate (4a). Synthesis of **4a** was similar to **3a** but using **2** as the platinum(IV) scaffold instead. Yield: ca.19 %; ESI-MS(-): m/z: 1237.1 $[M – 2H]^{2-}$; Purity (HPLC): 94 % at 280 nm.

Oxaliplatin(IV)-dAHNP conjugate (4b). Synthesis of **4b** was similar to **4a.** Yield: ca. 51 %. ESI-MS(-): m/z 1229.6 $[M – 2H]^{2-}$; Purity (HPLC): 96 % at 280 nm.

Cell culture. The HER2/neu over-expressing human gastric cancer cell-line NCI-N87 and breast cancer cell-line BT474 were obtained from ATCC. The A2780 human ovarian cancer cell line was obtained from EACC. All cell-lines were cultured in complete RPMI 1640 medium containing 10 % fetal bovine serum (FBS) and maintained in a humidified of 5 % CO_2 at 37 ^{o}C.

Annexin-V apoptosis assay (24 h). Apoptosis induced by drug treatment was assessed by double staining drug-treated cells with Annexin V-EGFP (abcam) and PI. Briefly, 2 x 10^5 cells were seeded per well in 12-well plates in complete media (1 mL) and allowed to adhere overnight. The test compounds were then diluted in complete medium and added to the cells at the concentrations indicated for 24 h. The old drug-containing media was then collected and the cells were harvested by trypsinzation and subsequently combined with the old media to deactivate trypsin. 2 x 10^5 cells per well were counted, pelleted and resuspended in 500 µL of Annexin-V binding buffer (10 mM HEPES/NaOH (pH 7.4), 140 mM NaCl and 2.5 mM $CaCl_2$) containing 1 x Annexin V-EGFP protein and PI (1 µg/mL). Samples were kept in the dark and analysed immediately on a flow cytometer (BD LSRFortessa). Quantitative analysis was performed using Summit software.

MTT assay for evaluating intermediate-term cellular metabolic activity (72 h). The indicated cell-lines were harvested from culture flasks by trypsinization and seeded at a density of 4.0 × 10^3 cells/well in 100 µL aliquots into flat-bottomed 96-well tissue-culture plates. Cells were allowed to adhere in drug-free complete media with 10 % FBS for 24 h, followed by the addition of dilutions of drug in 100 µL/well complete media and further incubated for 72 h. At the end of exposure, medium was replaced by 100 µL/well MTT solution (0.5 mg/mL in PBS). After incubation for 4 h, MTT was aspirated and substituted with 100 µL/well DMSO. UV-vis absorbance was measured at 570 nm using a microplate reader (Biotek). Experiments were

performed in sextuplicates for each drug concentration and carried out independently in triplicates. Metabolic activity was evaluated with the reference to the absolute IC_{50} value. IC_{50} values were calculated from concentration-response curves (cell viability against log of drug concentration) obtained in repeated experiments and adjusted to the actual concentration of Pt administered as measured by ICP-OES.

Clonogenic assay for evaluating long term reproductive viability (11 to 15 d). The cells were harvested by trypsinization into a single-cell suspension and subsequently the indicated number of cells, in complete media (2 mL), was seeded per well into a 6-well plate. Cells were allowed to adhere for 4 h, followed by addition of drug diluted to the indicated concentrations in complete media for 24 h. After 24 h, the cells were refreshed with fresh media (4 mL) and the cells was incubated for an additional 11 (BT-474) or 15 d (NCI-N87) to allow the single cells to recover and form colonies. The cells was then rinsed carefully with PBS (2 x 3 mL), fixed with 3 : 1 v/v MeOH: AcOH for 5 min, stained with crystal violet (0.5 % w/v ddH_2O) for an additional 15 min before rinsing thoroughly with water. Colonies containing greater than 50 cells were counted.

Drug targeting assay via co-culture of NCI-N87 and A2780. A2780 and NCI-N87 were cultured separately in T75 tissue culture flasks. A2780 was stained with CellTracker™ Green (2 uM) (Molecular Probes) in RPMI for 30 min. The media was aspirated and the cells were subsequently incubated with complete RPMI for a further 30 min. Both cell-lines were harvested by trypsinization and subsequently A2780 (2.5×10^4 cells / well) was co-cultured with unstained NCI-N87 (1×10^5 cells / well) in a 12-well plate. The cells were rested overnight before drug-treatment for 24 h. After 24 h, the old cell culture media (containing detached cells) was kept to deactivate the trypsin downstream. Trypsin-EDTA (300 µL) added to the adherent cells for 5 min and the harvested cells were transferred to the old culture media to deactivate (mostly A2780). Fresh trypsin-EDTA (300 µL) was added for a further 10 min and pooled together with the old culture media to deactivate (mostly NCI-N87). The combined cell fraction was washed with PBS (1 x 500 µL) and finally resuspended in PI (1 µg/mL) before analysis on a flow cytometer (BD LSRFortessa).

Intracellular platinum accumulation. For whole platinum accumulation, NCI-N87 (4×10^5 cells / sample) was drug treated for 4 h at 37 ^{o}C. After 4 h, the drug-containing media was aspirated and the cells were washed with PBS (2 x 500 µL). The cells was then centrifuged

down and the cell pellet was digested by heating with 65 % ultrapure HNO_3 (300 µL) at 90 °C overnight. The acid was boiled off and the cellular residue was re-dissolved by sonicating in 2 % HNO_3 (500 µL) for analysis by ICP-MS. Nuclear platinum accumulation was carried out as reported previously.[6] All values were reported as pmol per 10^6 cells.

Measurement of intracellular ROS. 2 x 10^5 cells was seeded in clear-bottom black 96 well plate and allowed to adhere overnight. After 24 h, the cells was washed with HBSS (1 x 100 µL) and prestained with 2',7'-dichlorodihydrofluorescein diacetate (H_2DCFDA) (15 µM in 100 µL HBSS) for 45 min at 37 °C. After washing with HBSS (1 x 100 µL), the cells were drug treated for the indicated duration. Drug were diluted in supplemented HBSS (containing 10 % FBS). At the indicated time and without washing, the fluorescence signal on the plate bottom (Ex: 485 nm / Em: 535 nm) was read using a microplate reader (Biotek), taking into account background fluorescence.

Cell cycle analysis. 2 x 10^5 cells were seeded per well in 12-well plates in complete media (1 mL) and allowed to adhere overnight. The test compounds were then diluted in complete medium and added to the cells at the concentrations indicated. At various time intervals, the culture media (containing detached cells) was removed and kept to deactivate trypsin downstream. The adherent cells was washed with trypsin-EDTA (500 µL) and fresh trypsin-EDTA (500 µL) was added to harvest the cells and the detached cells was then pooled together with the old culture media. The combined cell fraction was washed with chilled PBS (1 x 500 µL), followed by dropwise addition of chilled 70 % v/v EtOH/ water (500 µL) with constant mixing. After 1 h at 4°C, the fixed cells were washed with PBS (1 x 500 µL) and finally resuspended in RNase (MpBio, 500 µL of a 100 ug/ mL in PBS) and PI (50 ug/mL in PBS) for 1 h at r.t before analysis in a flow cytometer (BD LSRFortessa).

Visualisation of cellular morphology via fluorescence microscopy. 1 x 10^5 cells were seeded per well in 24-well plates in complete media (500 µL) on poly-L-lysine coated coverslips and allowed to adhere overnight. The cells were drug treated in complete media at the indicated concentrations for 24 h. After 24 h, the drug-containing media was replaced with fresh com. RPMI (1 mL). At various time intervals, the 90 % of the old media was aspirated, leaving just sufficient to cover the cells and fixed with an equiv.-volume of 3 : 1 v/v MeOH/ AcOH for 5 min. The fixative was carefully aspirated and fresh fixative (300 µL per well) was added for

another 10 min before washing with PBS (1 x 500 uL). Finally, the cells was stained with Hoescht 3342 (2 μg/ mL in PBS) for about 15 min at r.t and the coverslips was mounted onto slides for analysis on a confocal microscopy (FV1000, Olympus)

Supplementary Scheme

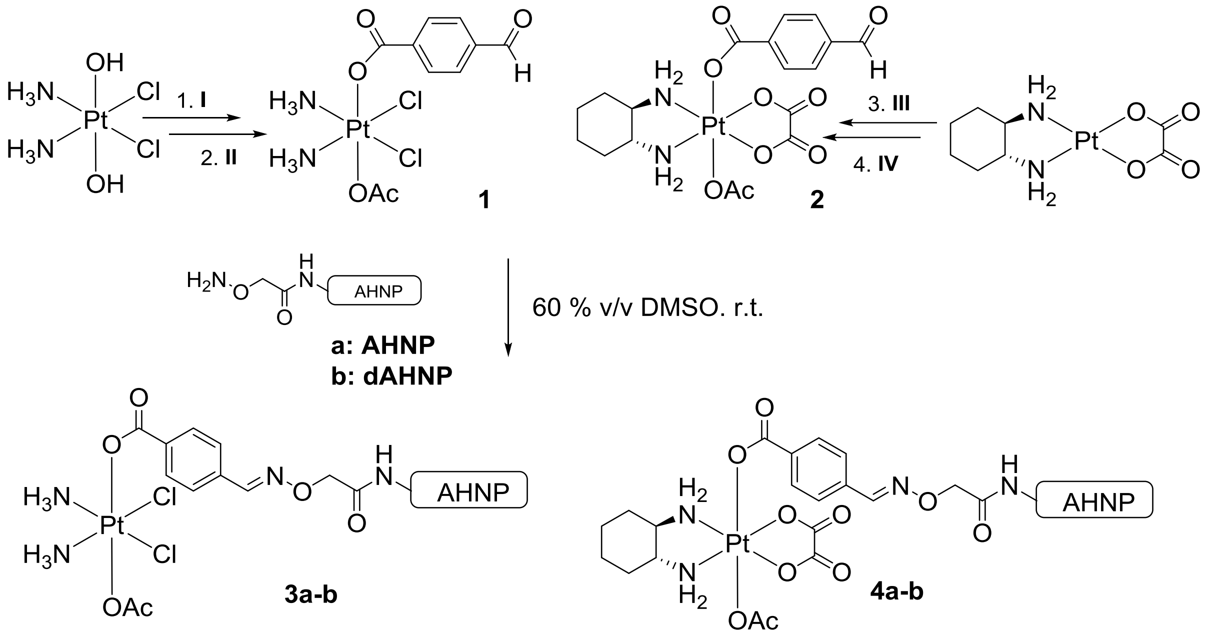

Scheme S1. Synthesis of platinum(IV)-AHNP conjugates. Reaction conditions: (I) succinimidyl 4-formylbenzoate, in DMSO, r.t. (II) acetic anhydride in DMF, r.t. (III) H_2O_2 in acetic acid, r.t. (IV) 4-formyl-benzoyl chloride in acetone, pyridine, reflux. AHNP: H_2N-YC*DGFYAC*YMDVGGKK(aminooxy)-$CONH_2$; dAHNP: fC*DGFYAC*yMDVGGKK(aminooxy)-$CONH_2$ (* - linked disulfide bridge).

Supplementary Figures

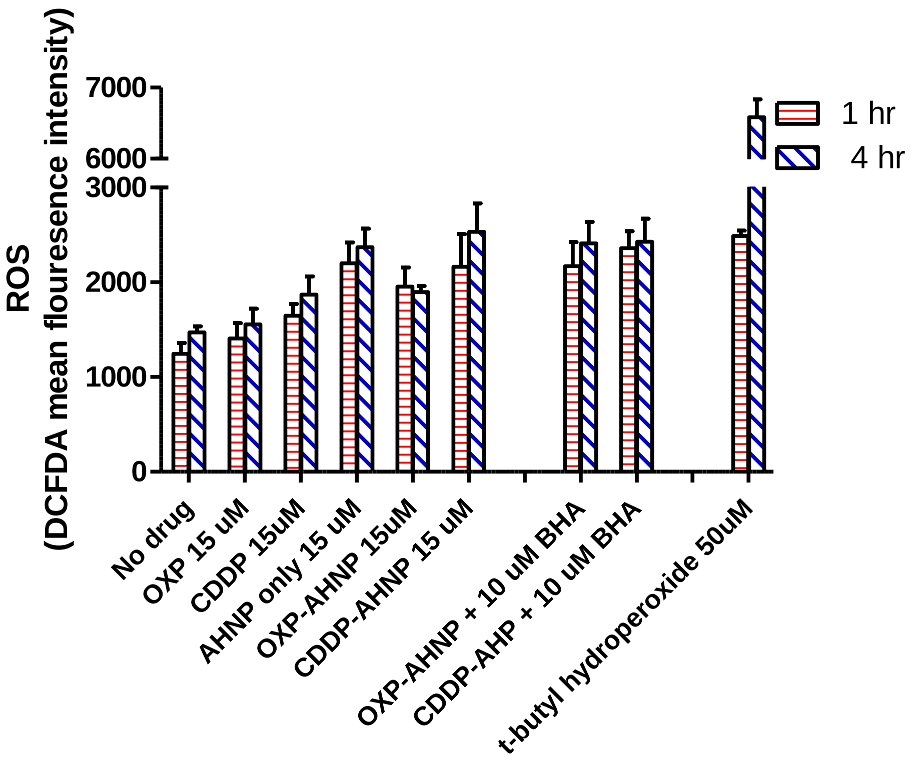

Figure S1. Measurements of cellular ROS on NCI-N87 after drug-treatment using 2',7'–dichlorofluorescein diacetate (DCFDA) which measures general oxidative stress. In some cases, cells were co-treated with butylated hydroxyanisole (BHA), an antioxidant. t-butyl hydroperoxide-treated cells was included as a positive control.

(a)

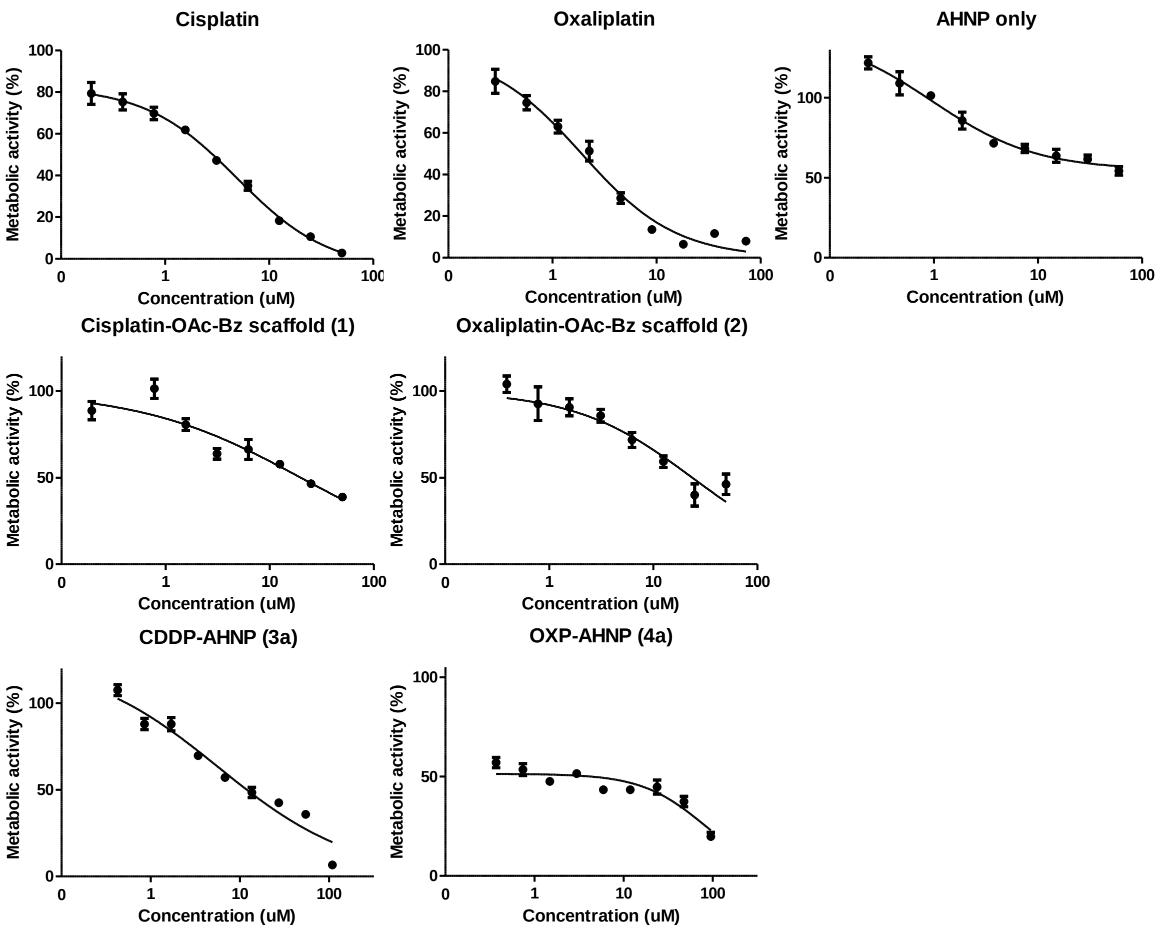

(b)

Compound	AHNP only	CDDP	1	3a	3b
IC_{50} (μM)	> 60	2.9 ± 0.3	23.1 ± 2.9	15.5 ± 2.4	> 100

Compound	OXP	2	4a	4b
IC_{50} (μM)	2.8 ± 0.6	21.8 ± 2.6	4.1 - 14.1	> 100

Figure S2. Intermediate-term potency of the drug conjugates as determined by MTT measurements of cellular metabolic activity after 72 h drug treatment against the NCI-N87 gastric cancer cell line. (a) Representative dosage-response curves (b) Table of absolute IC_{50} (μM). Platinum concentrations were calibrated by ICP-OES.

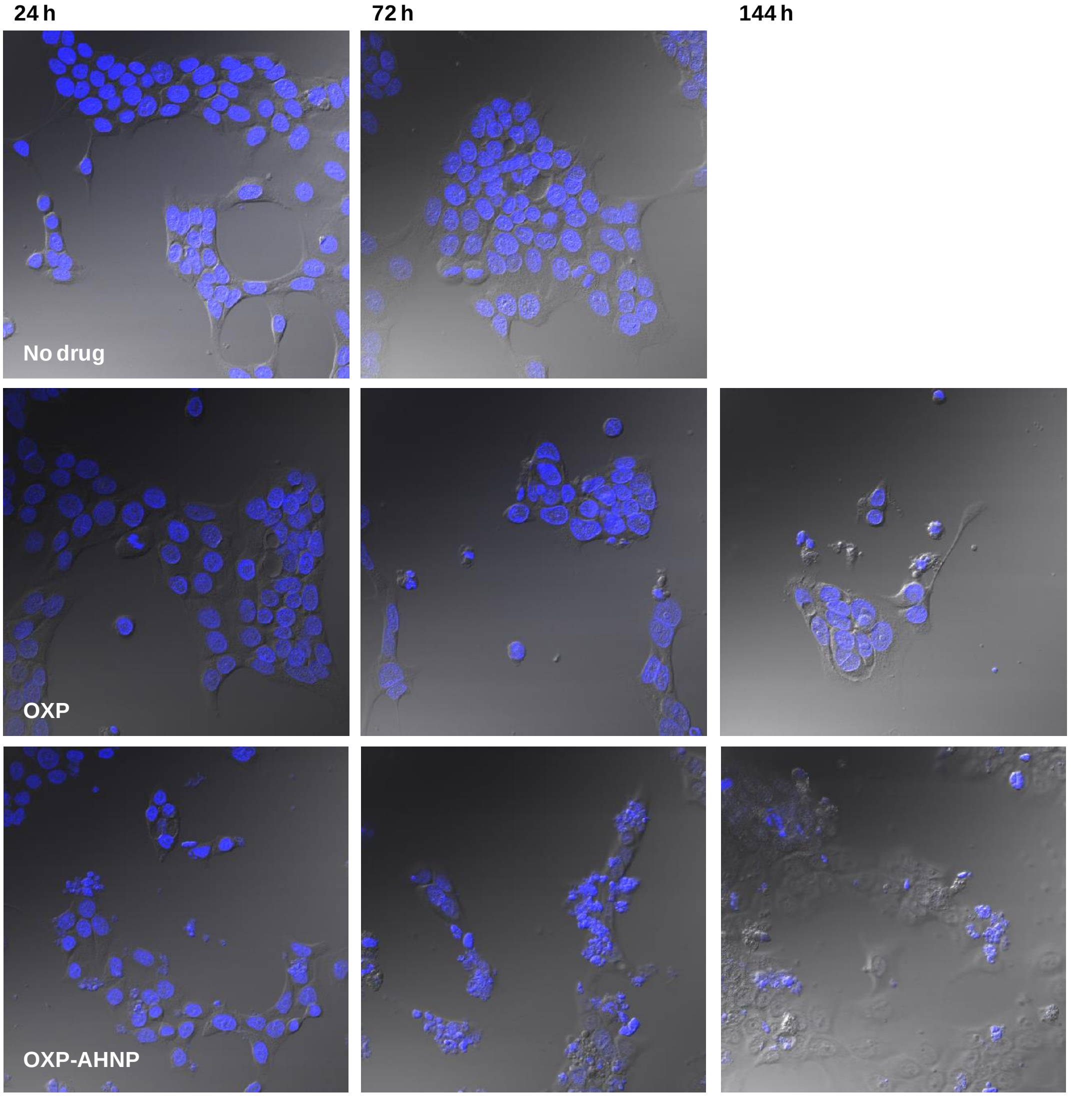

Figure S3. Visual flouresence microscopy monitoring of drug-treated NCI-N87 over time. NCI-N87 was drug-treated (15 μM for 24 h and then allowed to recover in fresh media). Nuclear morphology were visualized by Hoechst 3342 staining. There were substantial floating dead cells in drug-treated cells in the initial 24 h drug-treatment period (not shown here). When allowed to recover after 24 h drug-treatment, **4a**-treated cells undergoes a transient phase of cell-profileration, as evidenced by a decrease in empty space at 144 h. The cells exhibited increasingly abnormal nuclear morphology over time, implying that continued long-term profileration was unviable.

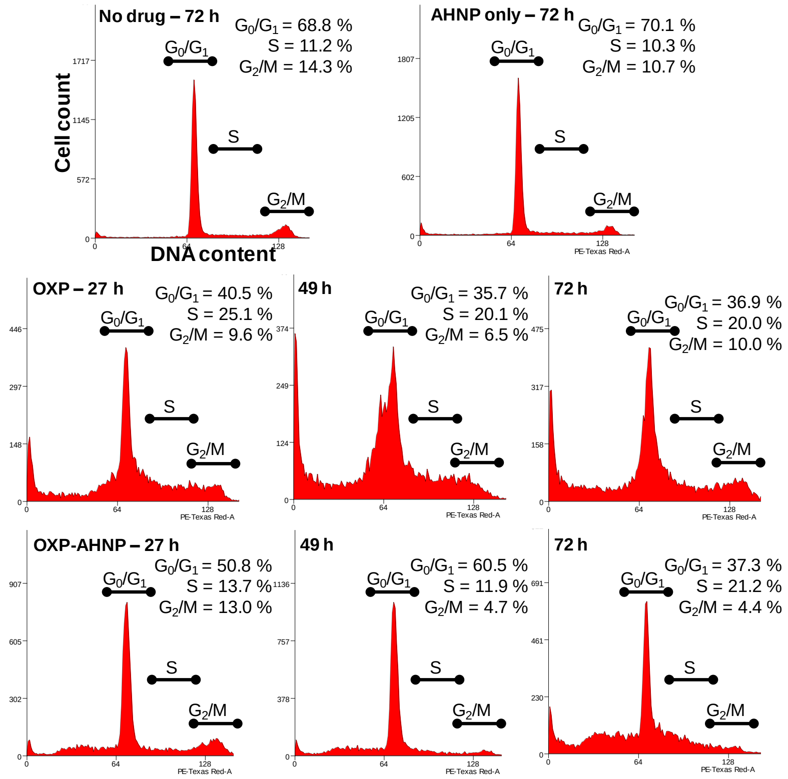

Figure S4. Cell-cycle distribution of drug-treated NCI-N87 cells over time. Drug-treated cells were permeabilized with 70 % v/v ethanol and stained with propodium iodide to determine DNA content.

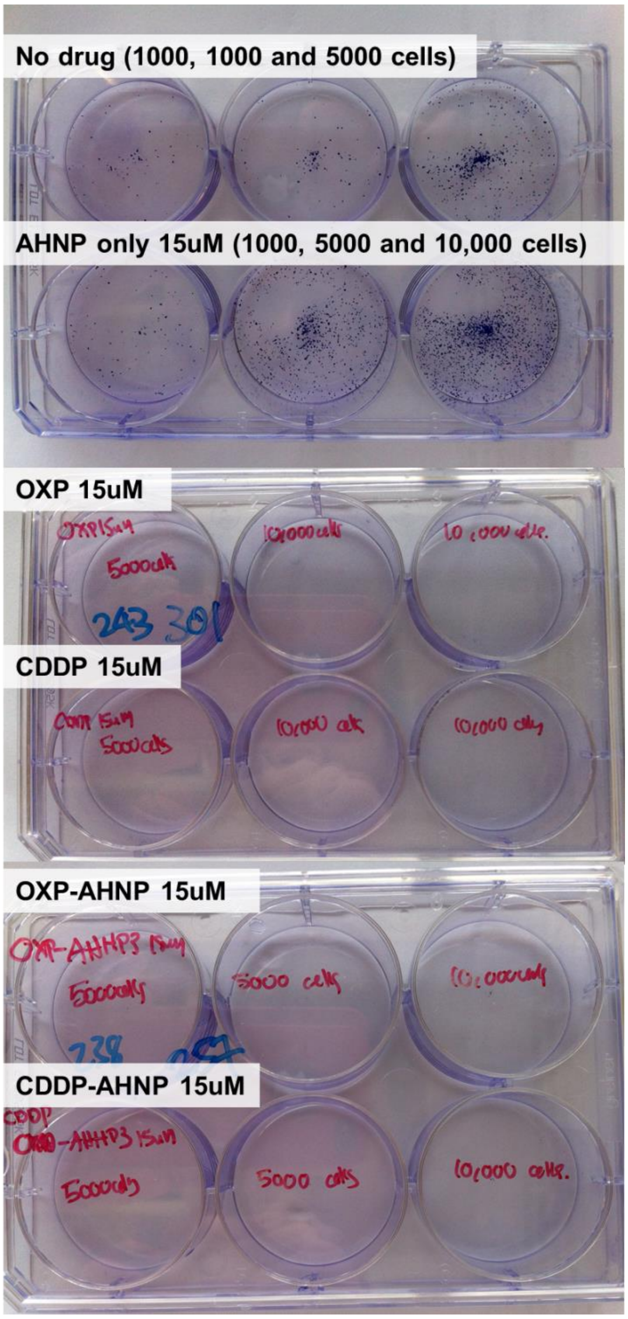

Figure S5. Representative photos of clonogenic assay on NCI-N87 to assess the long-term proliferation ability of drug-treated cells. Single cells were seeded in a 6-well plate, drug-treated for 24 h and allowed to recover in fresh complete media for a further 15 d before counting the number of colonies formed. The number of single cells seeded per well is written on the plates.

Characterisation of compounds

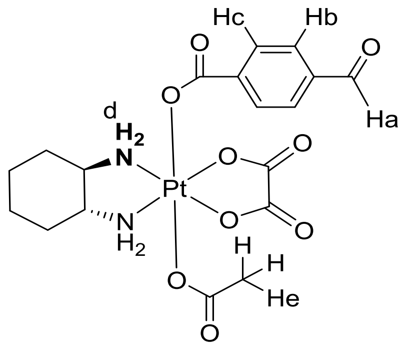

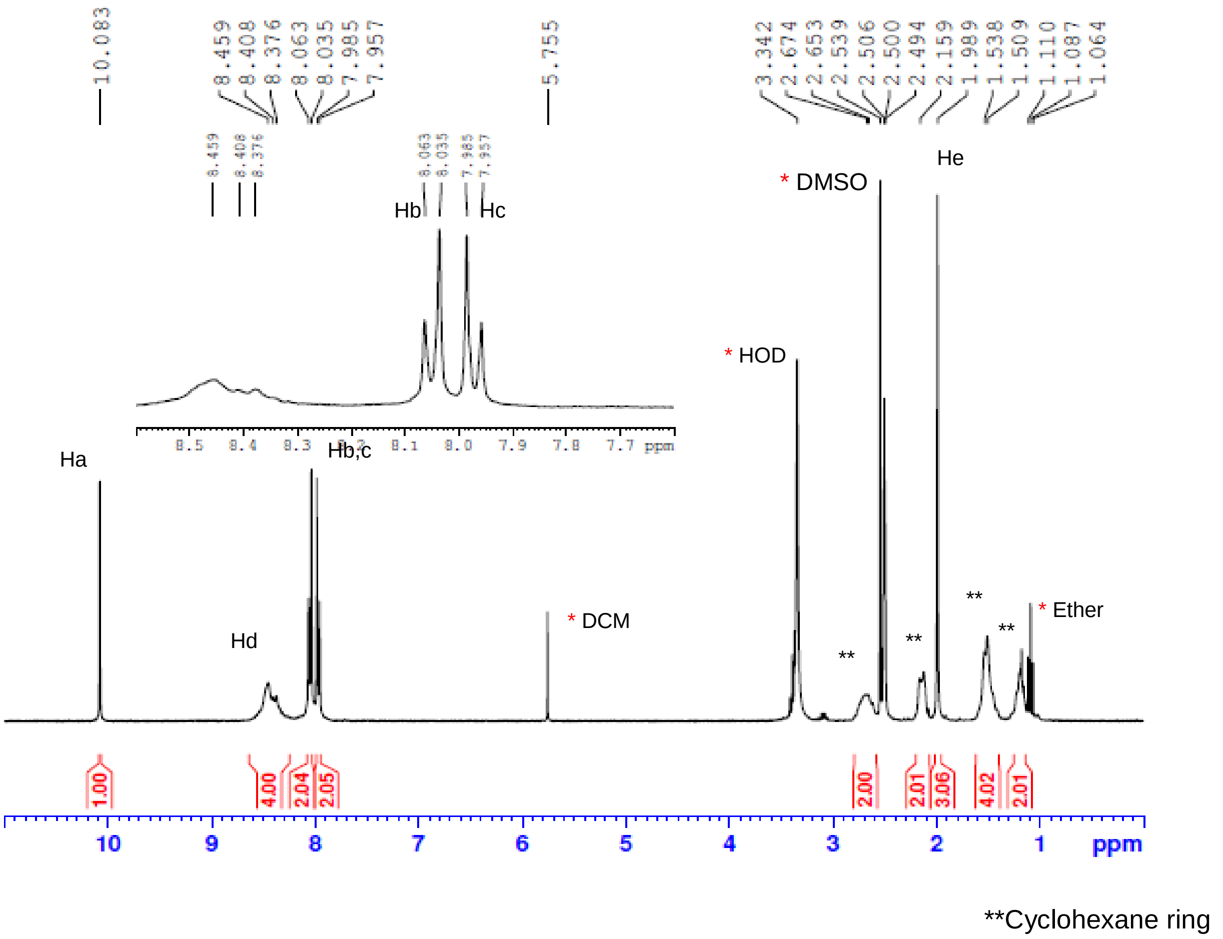

Figure S6a. ^{1}H NMR of oxaliplatin(IV)-benzaldehyde scaffold **2** in DMSO-d_6

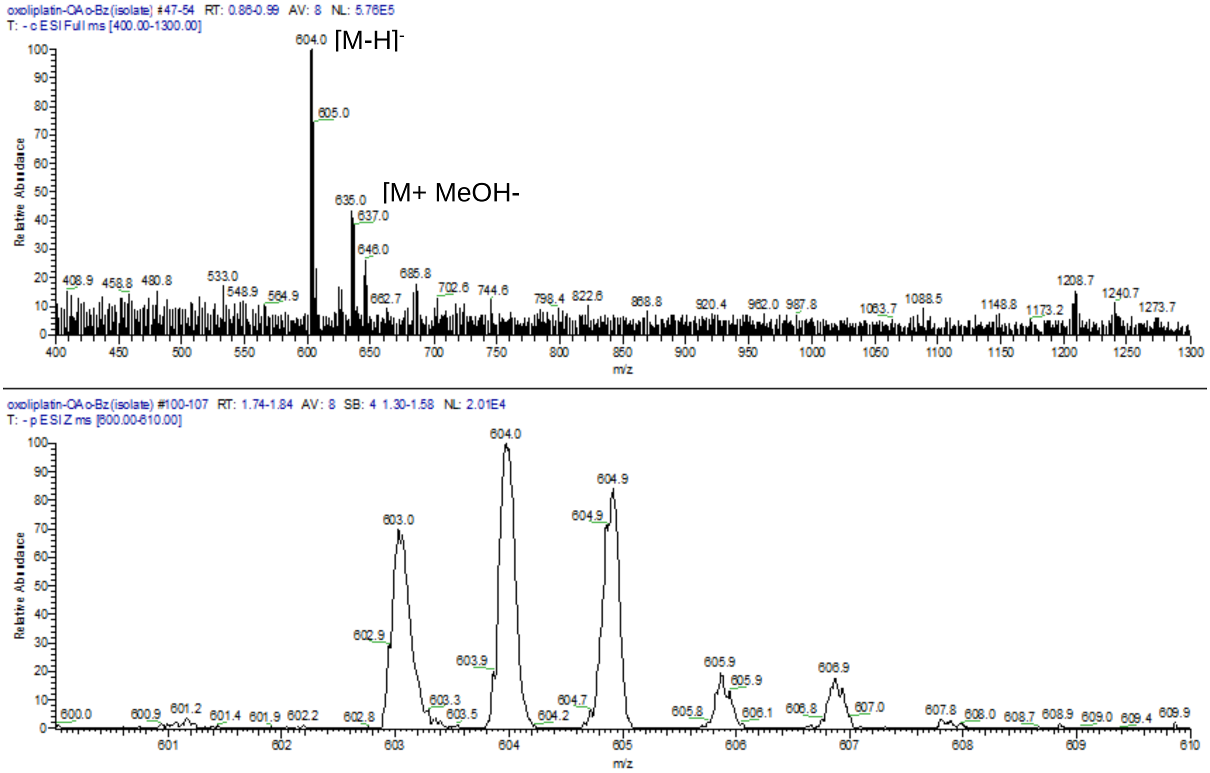

Figure S6b. ESI-MS (-) characterisation of **2**: Fullscan and zoom scan (isotopic pattern); m/z: calculated 604.5 $[M-H]^-$, found 604.0.

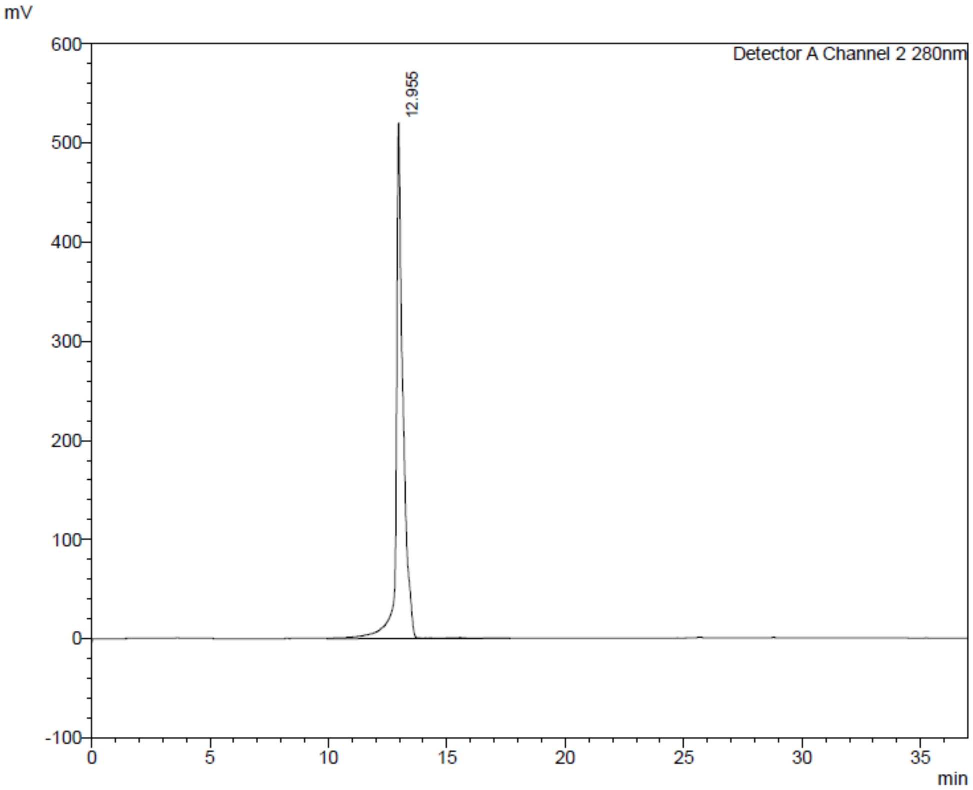

Figure S6c. RP-HPLC purity assessment of **2** dissolved in MeCN-H2O using Shimpack VP-ODS column (150 x 5.60 mm i.d). Elution condition: 8–30 % solvent B for 10 min, 30 % solvent B for 8 min and finally 30-80 % solvent B for 7 min.

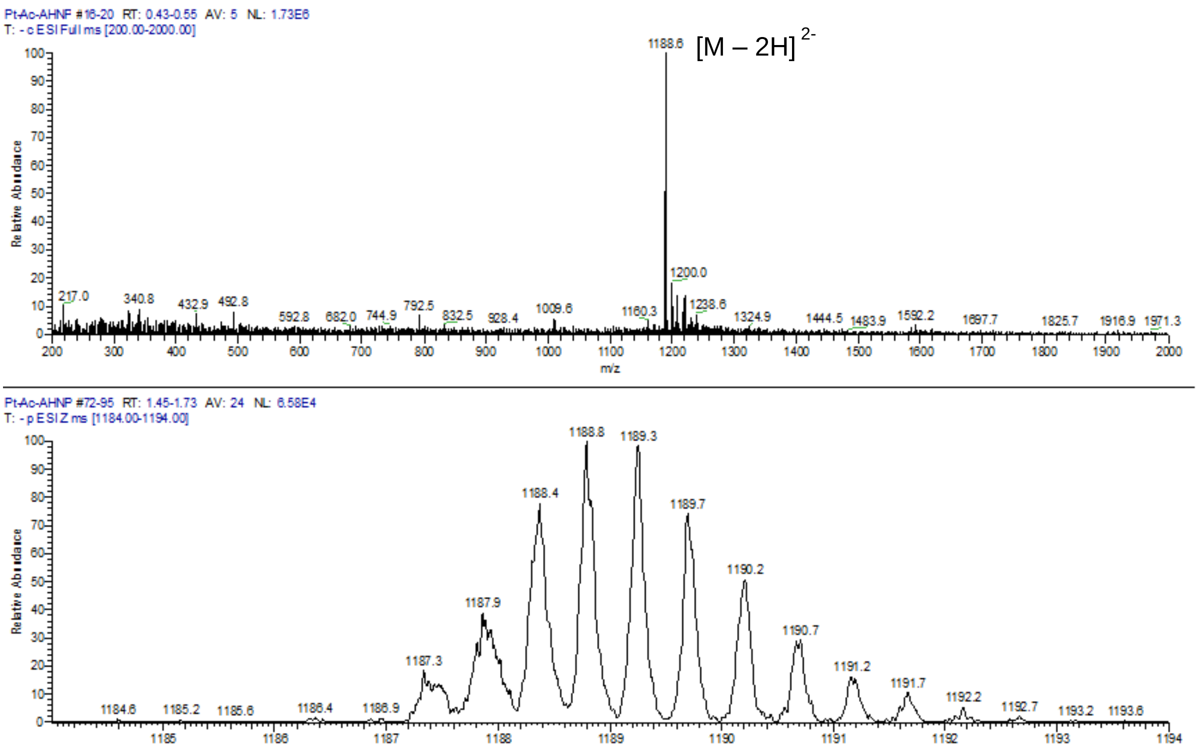

Fig. S7a. ESI-MS (-) characterisation of cisplatin(IV)-AHNP conjugate **3a**: Fullscan and zoom scan (isotopic pattern) m/z: calculated 1189.1 $[M-2H]^{2-}$, found 1188.6.

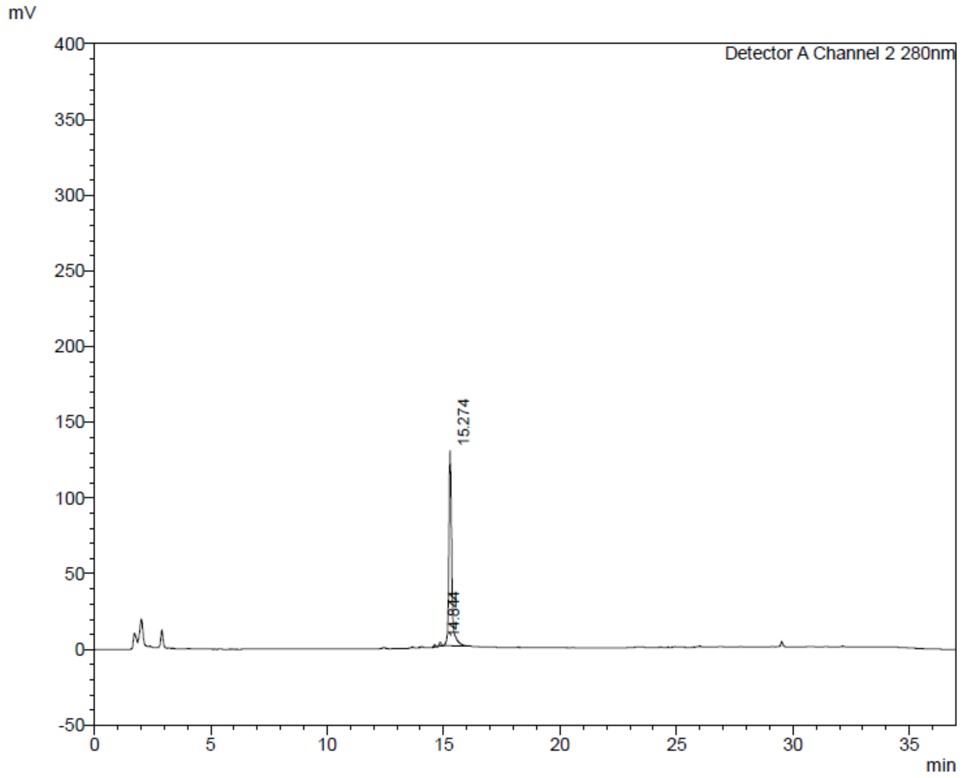

Fig. S7b. HPLC chromatogram of cisplatin(IV)-AHNP conjugate **3a**. The purity of the compound was assessed using Shimpack VP-ODS column (150 x 5.60 mm i.d). Elution Condition: 8–30 % solvent B for 10 min, 30 % solvent B for 8 min and finally 30-80 % solvent B for 7 min.

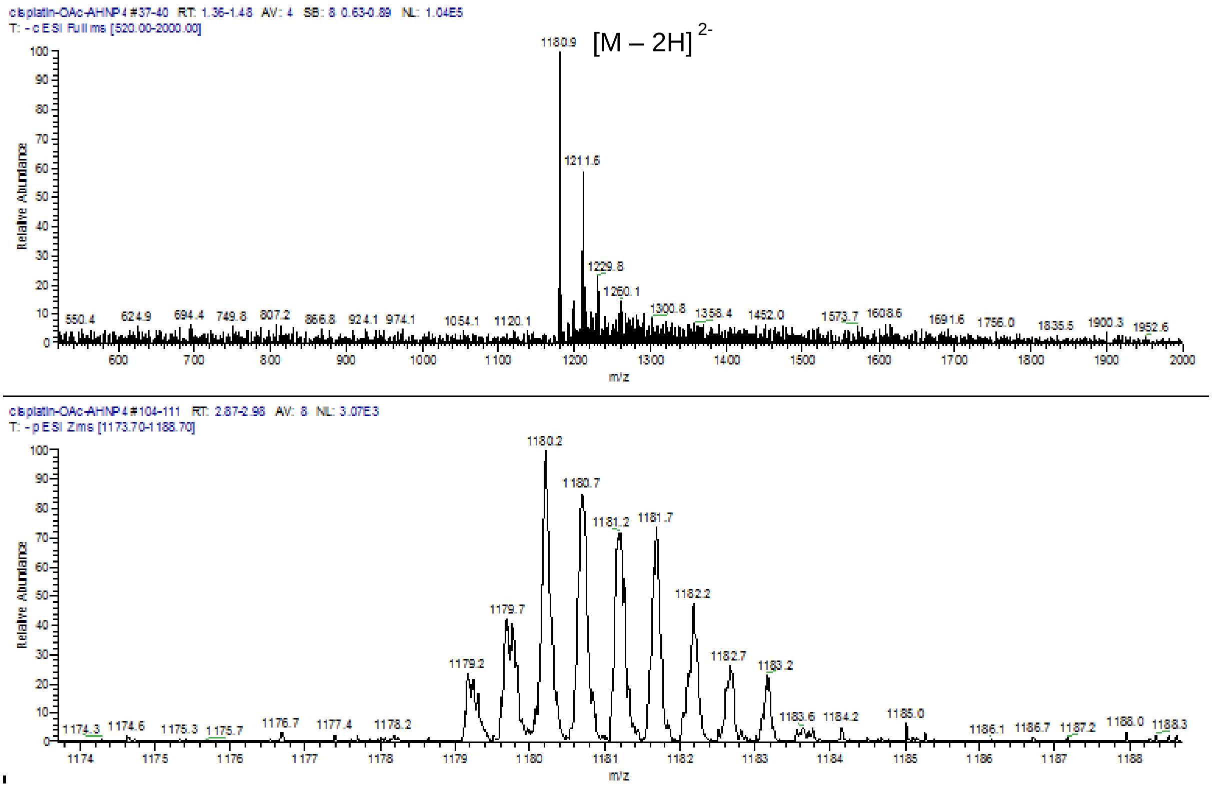

Fig. S8a. ESI-MS (-) characterisation of cisplatin(IV)-dAHNP conjugate **3b**: Fullscan and zoom scan (isotopic pattern) m/z: calculated 1181.1 $[M\text{-}2H]^{2-}$, found 1180.9.

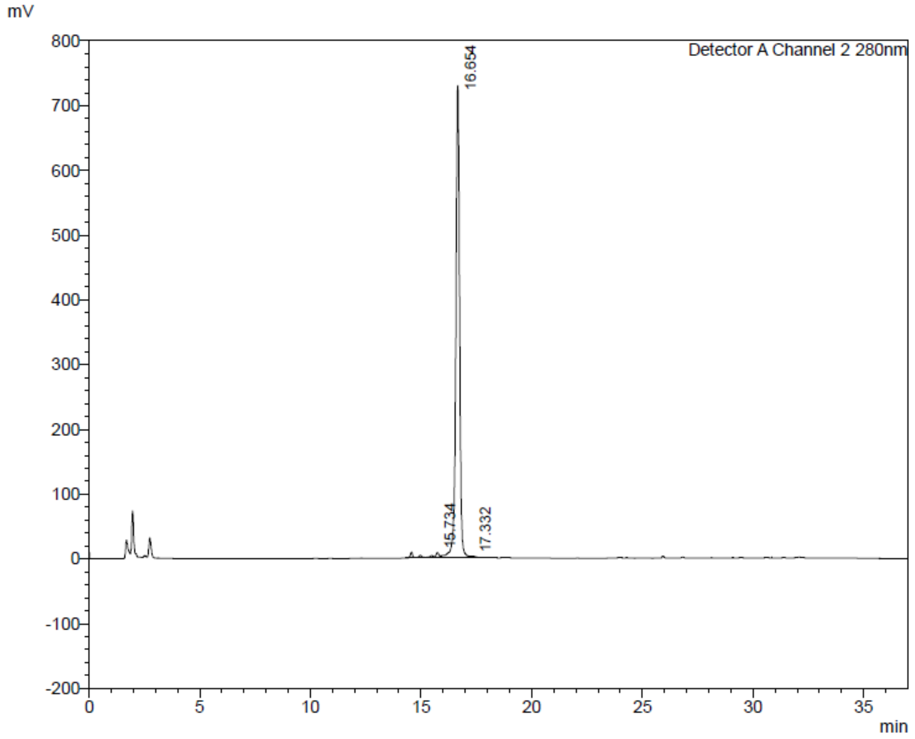

Fig. S8b. HPLC chromatogram of cisplatin(IV)-dAHNP conjugate **3b**. The purity of the compound was assessed using Shimpack VP-ODS column (150 x 5.60 mm i.d). Elution Condition: 8–30 % solvent B for 10 min, 30 % solvent B for 8 min and finally 30-80 % solvent B for 7 min.

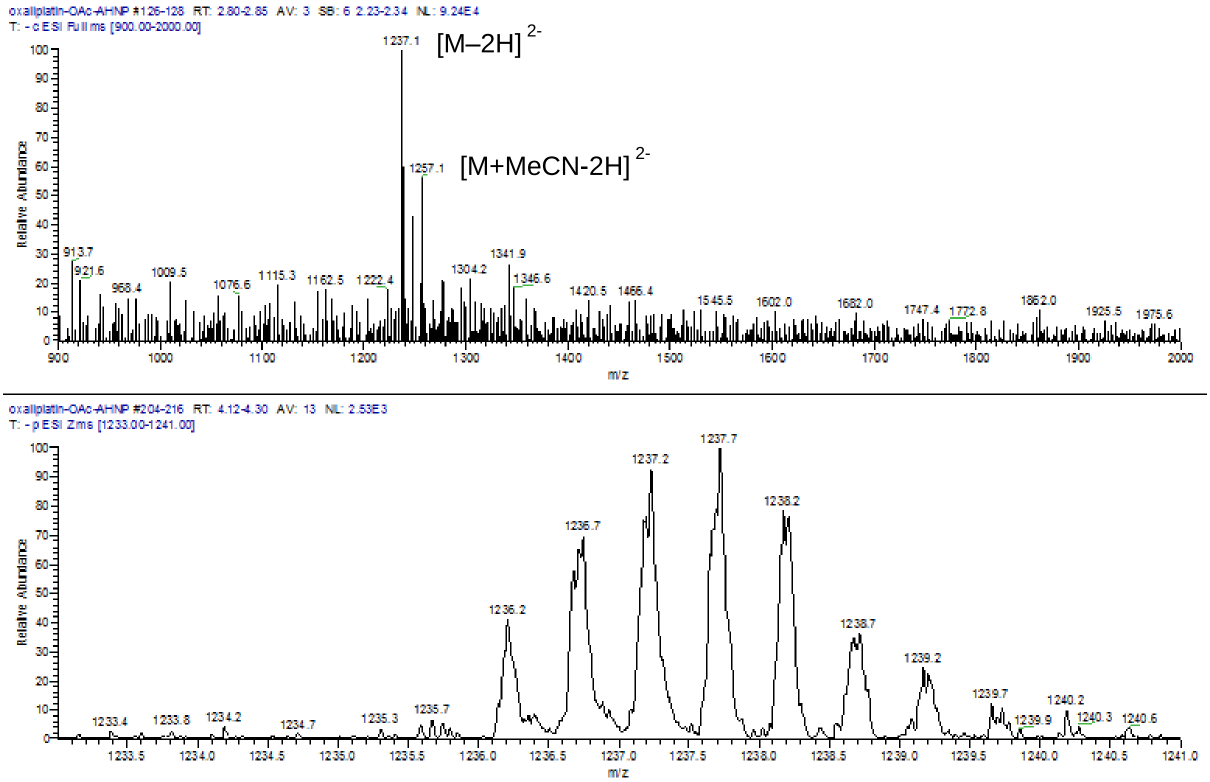

Fig. S9a. ESI-MS (-) characterisation of oxaliplatin(IV)-AHNP conjugate **4a**: Fullscan and zoom scan (isotopic pattern) m/z: calculated 1237.8 $[M-2H]^{2-}$, found 1237.1.

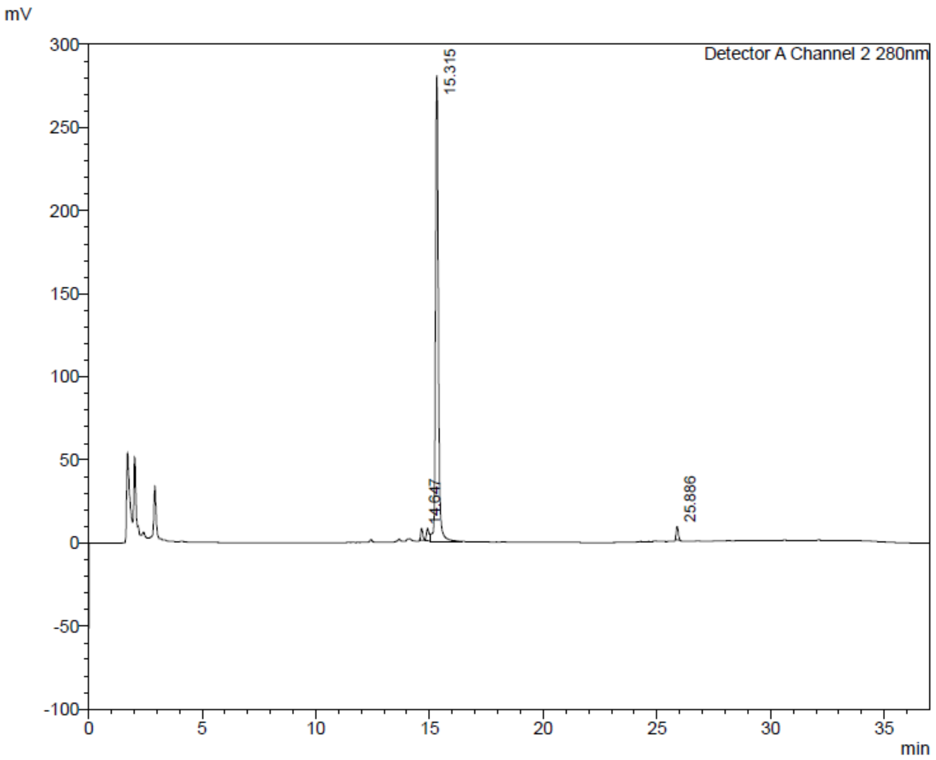

Fig. S9b. HPLC chromatogram of oxaliplatin(IV)-AHNP conjugate **4a**. The purity of the compound was assessed using Shimpack VP-ODS column (150 x 5.60 mm i.d). Elution Condition: 8–30 % solvent B for 10 min, 30 % solvent B for 8 min and finally 30-80 % solvent B for 7 min.

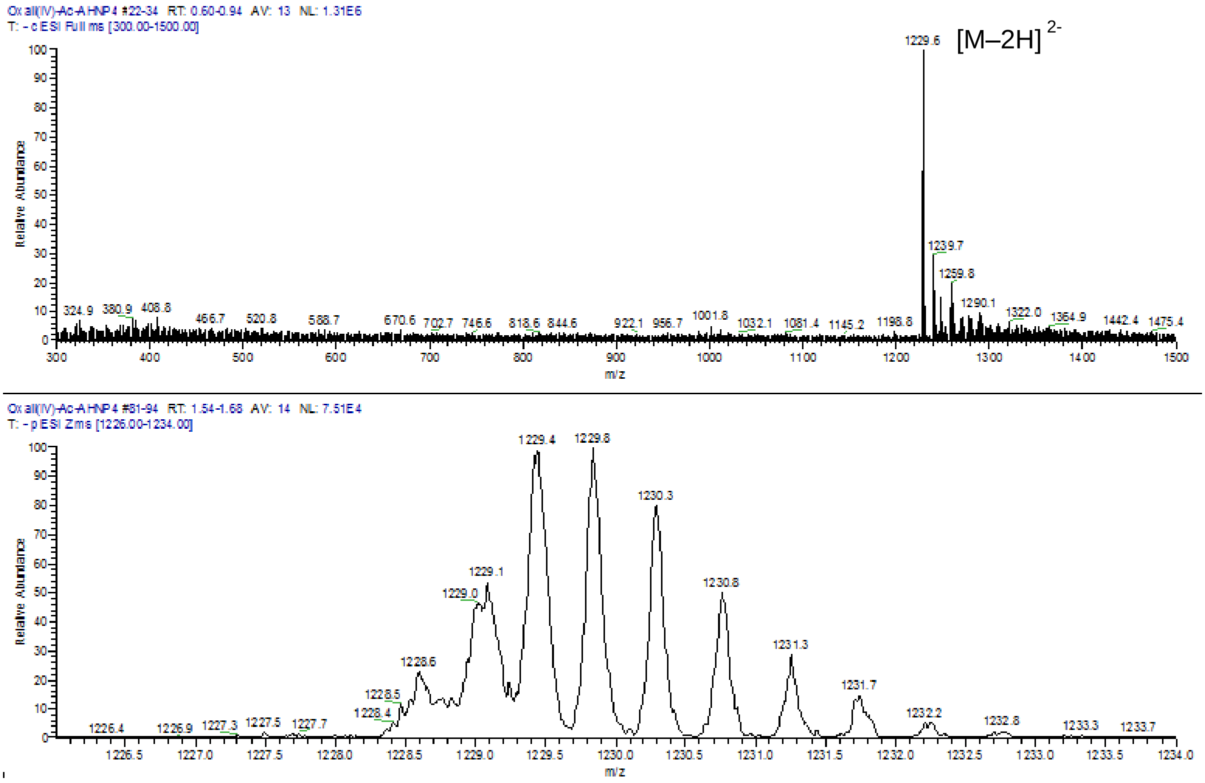

Fig. S10a. ESI-MS (-) characterisation of oxaliplatin(IV)-dAHNP conjugate **4b**: Fullscan and zoom scan (isotopic pattern) m/z: calculated 1229.8 $[M-2H]^{2-}$, found 1229.6.

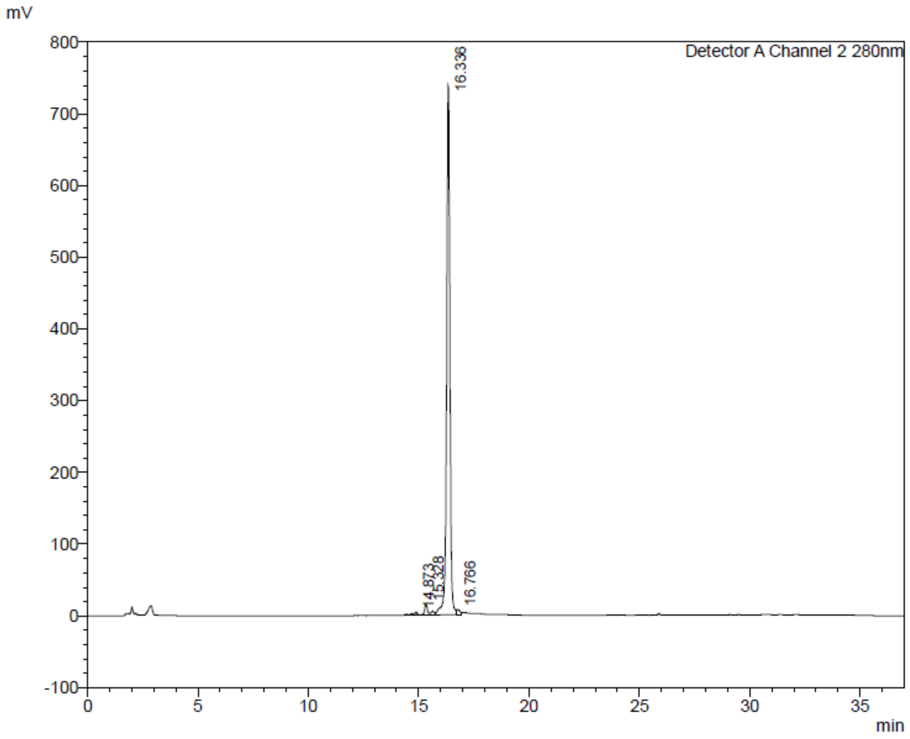

Fig. S10b. HPLC chromatogram of oxaliplatin(IV)-dAHNP conjugate **4b**. The purity of the compound was assessed using Shimpack VP-ODS column (150 x 5.60 mm i.d). Elution Condition: 8–30 % solvent B for 10 min, 30 % solvent B for 8 min and finally 30-80 % solvent B for 7 min.

References

(1) Wong, D. Y. Q.; Yeo, C. H. F.; Ang, W. H. *Angew. Chem. Int. Ed.* **2014**, *53*, 6752.

(2) Burgos, A.; Ellames, G. J. *J. Labelled Compd. Radiopharm.* **1998**, *41*, 443.

(3) Zhang, J. Z.; Bonnitcha, P.; Wexselblatt, E.; Klein, A. V.; Najajreh, Y.; Gibson, D.; Hambley, T. W. *Chem. Eur. J.* **2013**, *19*, 1672.

(4) Fulmer, G. R.; Miller, A. J. M.; Sherden, N. H.; Gottlieb, H. E.; Nudelman, A.; Stoltz, B. M.; Bercaw, J. E.; Goldberg, K. I. *Organometallics* **2010**, *29*, 2176.

(5) Gill, S. C.; von Hippel, P. H. *Anal. Biochem.* **1989**, *182*, 319.

(6) Park, G. Y.; Wilson, J. J.; Song, Y.; Lippard, S. J. *Proc. Natl. Acad. Sci* **2012**, *109*, 11987.
